# Intravital 3D visualization and segmentation of murine neural networks at micron resolution

**DOI:** 10.1038/s41598-022-14450-0

**Published:** 2022-07-30

**Authors:** Ziv Lautman, Yonatan Winetraub, Eran Blacher, Caroline Yu, Itamar Terem, Adelaida Chibukhchyan, James H. Marshel, Adam de la Zerda

**Affiliations:** 1 Department of Structural Biology, Stanford University School of Medicine, Stanford, CA 94305 USA; 2 Department of Bioengineering, Stanford University, Stanford, CA 94305 USA; 3 Molecular Imaging Program at Stanford, Stanford, CA 94305 USA; 4 Biophysics Program at Stanford, Stanford, CA 94305 USA; 5 The Bio-X Program, Stanford, CA 94305 USA; 6 Department of Neurology and Neurological Sciences, Stanford School of Medicine, Stanford, CA 94305 USA; 7 Department of Biological Chemistry, The Alexander Silberman Institute of Life Sciences, The Hebrew University of Jerusalem, Edmond J. Safra Campus Givat-Ram, 9190401 Jerusalem, Israel; 8 Department of Electrical Engineering, Stanford University, Stanford, CA 94305 USA; 9 CNC Department, Stanford University, Stanford, CA 94305 USA; 10 The Chan Zuckerberg Biohub, San Francisco, CA 94158 USA

**Keywords:** Applied optics, Biophysics

## Abstract

Optical coherence tomography (OCT) allows label-free, micron-scale 3D imaging of biological tissues’ fine structures with significant depth and large field-of-view. Here we introduce a novel OCT-based neuroimaging setting, accompanied by a feature segmentation algorithm, which enables rapid, accurate, and high-resolution in vivo imaging of 700 μm depth across the mouse cortex. Using a commercial OCT device, we demonstrate 3D reconstruction of microarchitectural elements through a cortical column. Our system is sensitive to structural and cellular changes at micron-scale resolution in vivo, such as those from injury or disease. Therefore, it can serve as a tool to visualize and quantify spatiotemporal brain elasticity patterns. This highly transformative and versatile platform allows accurate investigation of brain cellular architectural changes by quantifying features such as brain cell bodies’ density, volume, and average distance to the nearest cell. Hence, it may assist in longitudinal studies of microstructural tissue alteration in aging, injury, or disease in a living rodent brain.

## Introduction

The central nervous system (CNS) is comprised of thousands of neurons and glial cells, organized in a complex architecture that supports its functionality^1–3^. The exceptionally intricate nature of the brain posits a great challenge in studying its molecular signaling networks, biochemical processes, and cellular dynamics. In particular, it is difficult to isolate intact neurons for gene expression profiling due to their ramified, outspread, and fine-structured morphology. Recently developed methods utilizing tagged ribosomes, such as RiboTag, may allow further sorting-free characterization of neuronal subtypes^4^, however spatial information on cell–cell interactions, morphology, and tissue architecture is lost in the process of isolating actively translated mRNA.

Therefore, advanced neuroimaging techniques are necessary for integrated research platforms that combine functional, temporal, and spatial dimensions into a comprehensive brain study’s methodology. Recently, intravital multiphoton microscopy imaging of mouse strains expressing a *Cre*-dependent calcium indicator (GCaMPs) has been leveraged to explore neuronal signal transmission *in vivo*^5^. Tissue-clearing methods such as CLARITY^6^ and iDISCO^7^ are instrumental for 3D reconstruction of complex neuronal networks ex vivo, while DREADD^8^ and optogenetics^9^ are effective for in vivo neuronal activities’ modulation. However, key challenges remain: 1) invasive administration of labeling contrast media, 2) dependency in genetically modified animals expressing *Cre*-driven elements or reporter proteins, and 3) incompatibility of most of the existing modalities with in vivo longitudinal imaging. These limitations may result in an expensive and time-consuming process for in vivo imaging of a limited range of targets. Accordingly, novel imaging tools are needed for in vivo imaging at cellular resolution allowing wide coverage of regions of interest (ROI) and spatiotemporal monitoring of cells in a minimally disrupted biological context.

Optical coherence tomography (OCT) – a fast, wide-field, label-free, deep-penetrating neuroimaging tool (up to ~ 1.5 mm), with a micron-scale spatial resolution – may meet the criteria stated above. OCT is an interferometric imaging microscopy allowing for 3D tomography of the tissues’ scattering properties at micron resolution. It is mainly used in ophthalmology^10–13^ and in vascular and hemodynamics research^10, 14^, with some implications in dermatology^15, 16^, dentistry^17, 18^, and cancer diagnostics^19–23^. Applying OCT to neuroimaging, with or without contrast agents, is gaining increased recognition^24–26^.

Nevertheless, OCT has some disadvantages, e.g., the lack of cell-type specificity, and the challenge of filtering out noise from background scattering. With these in mind, we set out to longitudinally track neuroanatomical fine structures, such as cellular CNS structural dynamics, and to quantify morphological biological outputs in a living mouse brain. Previous studies utilizing optical coherence microscopy (OCM) demonstrated large imaging depth and high-resolution *en face*, with a relatively small field of view^27, 28^. Other OCT-based studies have achieved either high-resolution *en face*^29^ or a significant imaging depth^30^. Here we demonstrate a straightforward OCT-based imaging acquisition protocol coupled with a feature segmentation algorithm that enables imaging of CNS cell bodies and micro-tractography of axons in a wide-field cortical column at a micrometer-scale resolution. Using a cranial window, we were able to image 700 μm of the cortex (see Supplementary Media 1). To the best of our knowledge, this is the first in vivo OCT study that demonstrates large imaging depth, high-resolution *en face*, and a large field of view in one bend, while using a commercially available OCT platform. Therefore, this approach may serve as a compelling technique to simultaneously track multiple cellular behaviors in living rodents for a wide range of basic and pre-clinical research needs, such as traumatic brain injury, stroke, and neurodegeneration.

## Results

### Deep OCT volumetric imaging of murine visual cortex at a cell-scale resolution

**Figure 1:**
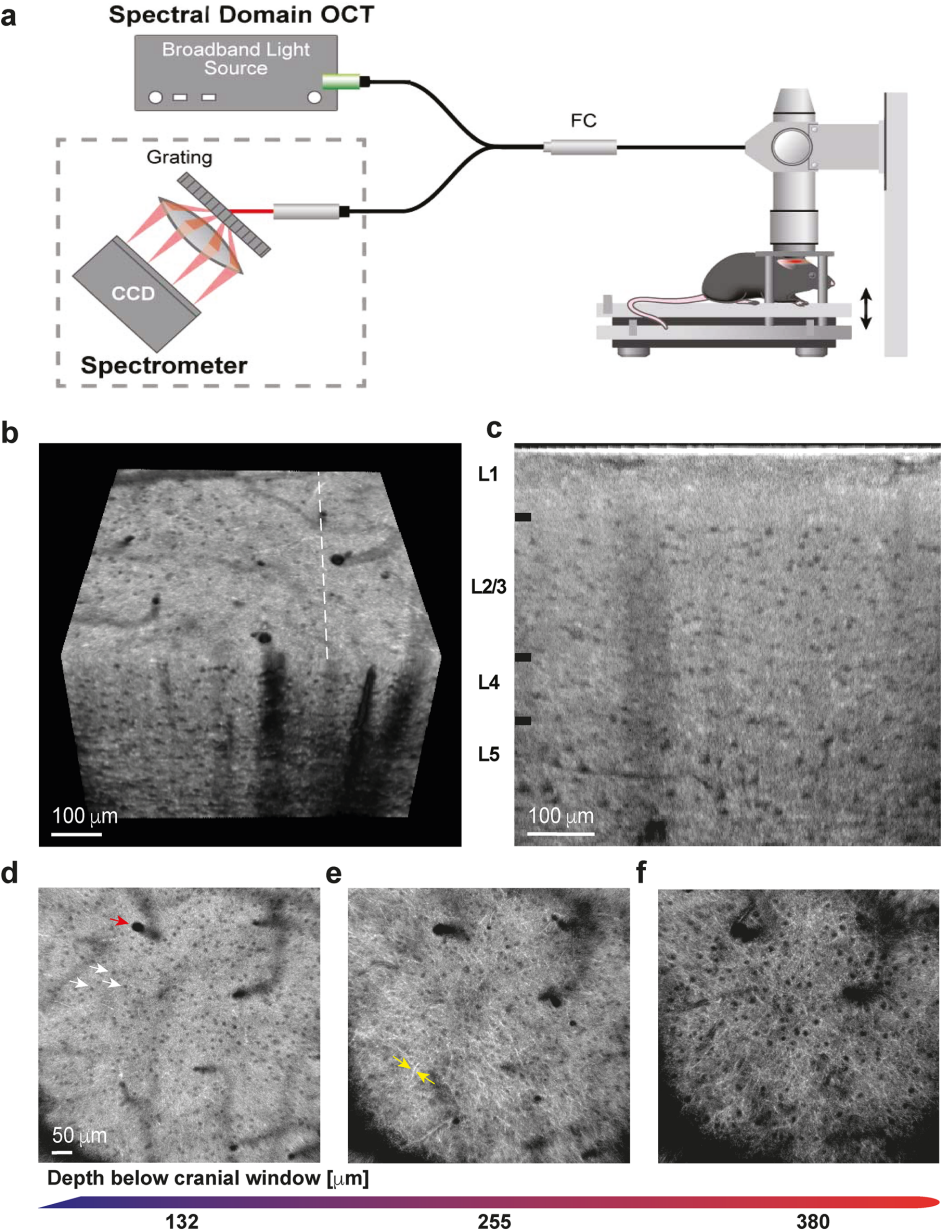
High-resolution cortical imaging by highly stable OCT system in vivo. (**a**) Schematic of our OCT imaging setup showing fixation of a mouse head by a customized head-clamp that is mounted on a translational (z-direction) stage. (**b**) A sampled 3D OCT volume (0.7 X 0.7 X 0.4 mm) of the mouse visual cortex (**c**) Sampled OCT b-scan, at the location marked by the dashed line in panel (**b**), of a cross-section of the five cortical layers (marked L1-L5) in the mouse visual cortex. Cell bodies appear as circular dark foci. (**d–f)** Sampled high-resolution *en face* images at cortical depth (**d**) 132 µm, (**e**) 255 µm, and (**f**) 380 µm [contrast in (**d**–**f**) was manually enhanced to emphasize cell bodies]. Red arrow points to a blood vessel, the white arrow points to CNS cell bodies, and the yellow arrow points to a myelinated axon.

Our main objective in this study was to develop a protocol for a robust, deep-penetrating, labeling-free, reproducible cortical imaging of the mouse brain at a cellular-scale to enable the characterization of tissue morphology in a large field of view. To this end, we utilized a commercial OCT (Thorlabs GAN220), outfitted with a 20 × water immersed Olympus lens (UMPLFLN20XW). We achieved a highly stable imaging configuration by fitting the mouse into a customized head clamp device^31^, while placed on a translational Z stage (Fig. 1a). To image a cortical column in vivo, open-skull cranial windows were installed as previously described^31^. Three month old C57/BL6J mice were anesthetized and a circular (1 cm diameter, 1 mm height) titanium implant with a counterbore (8 mm outer diameter and 4 mm through-hole) was affixed to the skull with Metabond dental cement (Parkell) centered at − 2.75 mm (lateral) and − 2.25 mm (posterior) from bregma over the left lateral primary visual cortex. Our spectral-domain OCT system (center wavelength of 900 nm and 200 nm bandwidth) provides a theoretical axial resolution of 2 μm (FWHM) in tissue and a theoretical lateral resolution of 0.76 μm (FWHM) with an effective numerical aperture of 0.5.

Mice were then placed on an XYZ translation stage (Thorlabs MT3-Z8) for multiple volume imaging. The interval between two consecutive focal plains was set to 10 μm (Fig. 1a). This approach allowed us to overcome the lens’s limited ~ 11 μm depth of field. Notwithstanding, stabilizing the mouse to be motionless throughout the entire imaging session (~ 40 min) posited a substantial technical challenge (see “Methods”).

Next, we used an algorithm developed in our lab to computationally stitch the imaged volumes in a way that preserves focus throughout the entire field of view (Fig. 1b)^32^. We achieved a conserved isotropic lateral resolution of 1.1 μm throughout the 700 μm depth of the cortical column across a large field of view of up to 0.7 × 0.7 mm (see Supplementary Media 1). This enabled us to reconstruct high-resolution 3D imaging of the mouse visual cortex, including cellular morphologies, such as myelinated axons, and neuronal cell bodies across cortical layers I–V (Fig. 1c). Notably, we focused our analysis on cortical layers I–V. CNS cell bodies appeared as circular dark foci, while myelinated axons processes manifest as white ramifications protruding across the cortical layers (Fig. 1d–f, Supplementary Media 1). These structural appearances were previously demonstrated by co-registering two-photon microscopy images and immunohistochemistry of the same brain region in mice^27^ and rats^28^. In addition, similar OCT-gained structural appearances have been validated by histopathology in humans^33, 34^. Our results recapitulate a more recent study that compared in vivo OCM imaging of the mouse brain with histology^35^. Furthermore, similar structural findings have been reported by others^29, 36–38^. These results indicate that we successfully introduced a new robust way to utilize a commercially available OCT system for a wide coverage of endogenous high-resolution cellular imaging of fine structures in a living mouse, across five cortical layers.

### Feature segmentation algorithm harnesses neural network computability

**Figure 2:**
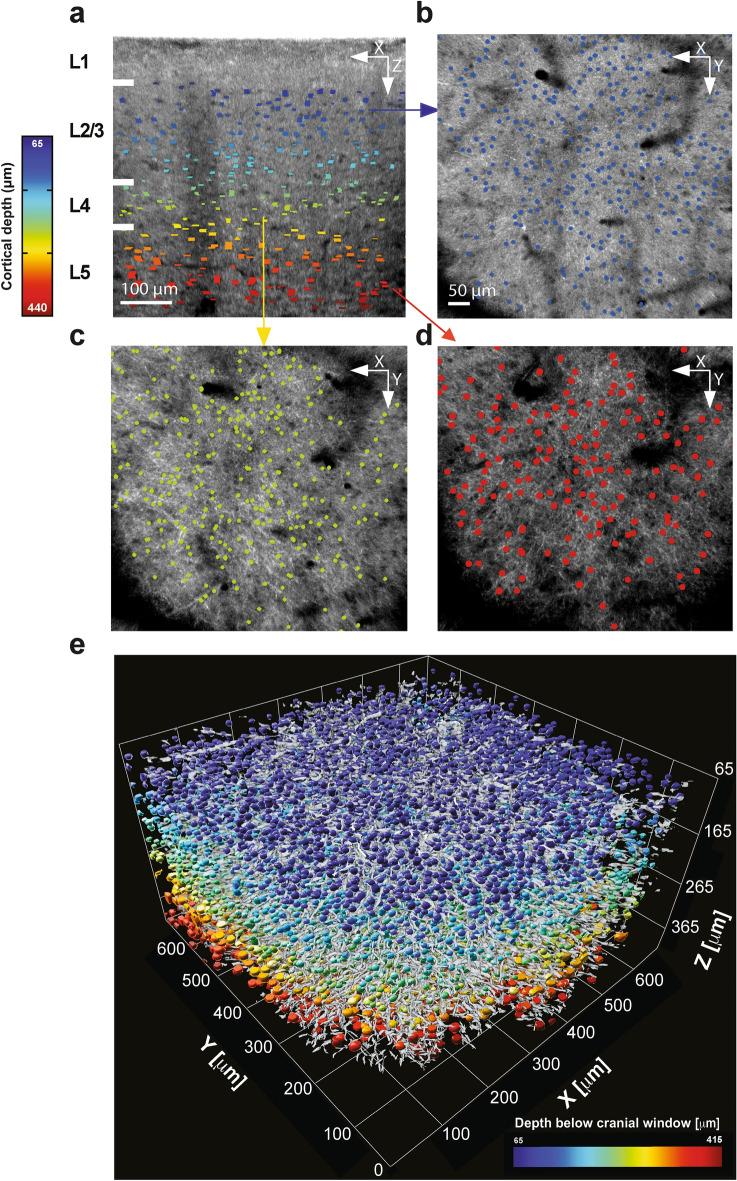
Feature segmentation method allows for micron-scale 3D visualization of the cortex. (**a**) A sampled b-scan overlaid with the segmented CNS cell bodies; color encodes depth below the cranial window (**b–d**) Sampled *en face* images overlaid with the segmented CNS cell bodies at cortical depth (**b**) 132 µm, (**c**) 255 µm, and (**d**) 380 µm. (**e**) Visualization of a 3D CNS segmentation mask of brain neural networks in a living mouse at micron-level resolution, with a color-encoded depth of more than 10,000 CNS cell bodies, and their surrounding myelinated axons (grey); volume dimension is 0.7 X 0.7 X 0.4 mm (XYZ).

To gain more biological insights from our imaging platform, we developed a feature segmentation algorithm and applied it to our imaged cortical volumes. This algorithm is based on one layer convolutional neural network principles^39^. This is an additional original aspect of our work, demonstrating how leveraging neural network approaches can be a highly effective strategy for cell segmentation. As such, individual *en face* slices were convolved with a circular kernel (see Supplementary Fig. S1). Following convolution, we applied 3D morphological thresholding to reconstruct a 3D CNS cell body mask using three conditions (see Supplementary Fig. S1): (1) A cell body must appear in at least three consecutive axial slices, i.e., having a minimum depth of 4 µm and a maximum depth equal to its diameter, (2) a cell body’s volume should not exceed the volume of a sphere with an equal radius, and (3) its axial spread should not exceed twice its diameter. The latter was applied to exclude segmentation of blood vessels, which can also appear as circular dark foci (Fig. 1d). The kernel diameter was manually adjusted for each cortical layer by averaged cell size (see Supplementary Fig. S2). To image cortical layers II-IV we used a circular 8 μm diameter kernel, and a 13 μm diameter kernel for layer V (see Supplementary Fig. S2). This enabled us to segment CNS cell bodies across cortical layers II-V (Fig. 2a–d). Layer I was excluded from further analyses since it has sparser neurons compared to other layers and suffered from light aberrations from the cranial glass window.

**Figure 3:**
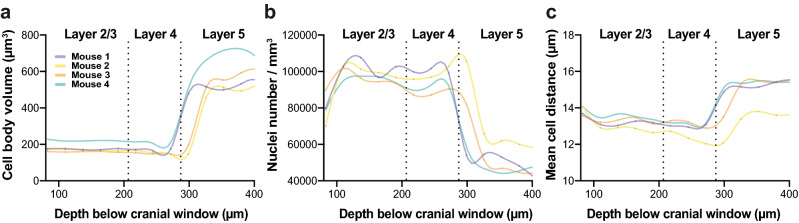
Quantifying cellular morphological features across the cortical column of mice in vivo. Four male mice were imaged by our OCT neuroimaging system and morphological traits were assessed and quantified: (**a**) average CNS cell body volume (**b**) average CNS cell body density (**c**) average distance to the nearest neighboring CNS cell body.

Next, we sought to add a myelinated processes’ segmentation capability into our feature segmentation tool, which appeared in OCT as white filaments^27, 28, 33, 36, 38^. For this purpose, we applied a modified cell body segmentation algorithm, using four features representing vertical, horizontal, and two diagonals’ filaments-like structures. Imaging CNS cell bodies and myelinated processes segmentation in 3D by our system, revealed a complex interconnected neural network across the cortical column (Fig. 2e, Supplementary Media 2). Of note, OCT mainly reveals myelinated processes oriented perpendicularly to the optic axis, perturbating along the *en face* plane^27, 28^. Importantly, a 3D CNS cell body mask allowed us to quantify the average cell body density, average volume, and average distance to the nearest neighboring cell for each segmented volume (Fig. 3a–c), in a 27 μm-thick increment block. It is worth noting that our data of cell bodies’ density align with other reports that performed histological validations^40–43^. Although some intra-individual variability may be noted, these three parameters were reproducible across our biological repeats (Fig. 3). Taken together, these results suggest that our imaging acquisition system, followed by a segmentation method, may serve as a new strategy to spatially explore the microstructure of CNS cells and myelinated processes across a cortical column.

### Deep OCT brain imaging allows temporal resolution for longitudinal studies

**Figure 4:**
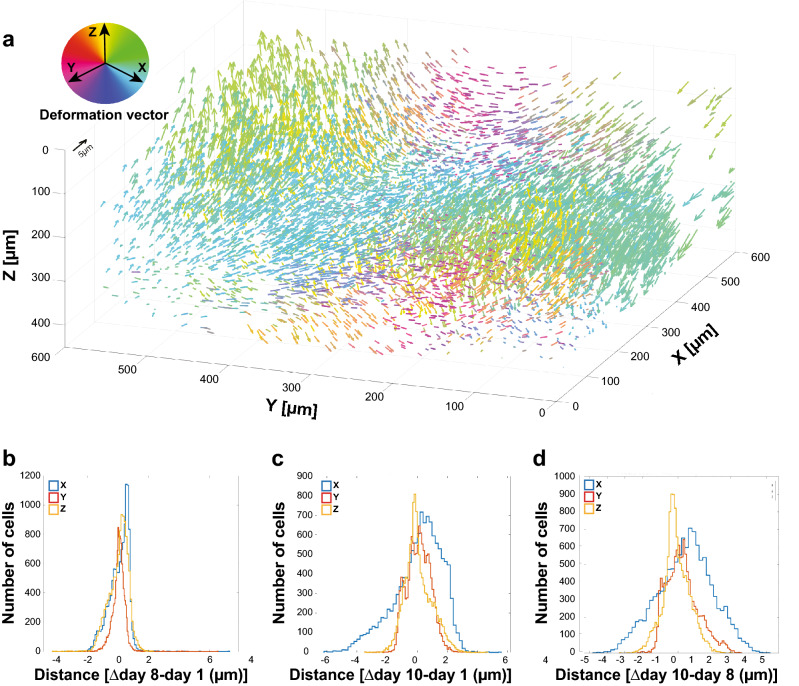
A cortical elasticity map assesses temporal cellular structure dynamics in vivo. (**a**) CNS cell bodies’ positional temporal changes, attributed to subtle tissue movement, between Day 1 and Day 10 of imaging as a function of the difference between rigid and elastic registration. Arrow’s color represents the spatial positional temporal changes, based on each individual cell body location change, while its length indicates the distance. (**b–d**) spatial positional temporal changes (distance) histogram of the center position offset of registered CNS cell bodies across X, Y, and Z axes between time points. The histogram shows only registered cells that were less than 10 µm apart, between (**b**) imaging Day 8 to Day 1 (76% of cells), (**c**) imaging Day 10 to Day 1 (76% of cells), and (**d**) imaging Day 10 to Day 8 (78% of cells).

To further explore CNS cell body resolution structural dynamics longitudinally, we imaged the same mice, at the same field of view, across three different time points (Days 1, 8, and 10). To accurately revisit the same ROI, we applied a rigid-body registration, followed by an elastic one. First, we applied a course registration by matching blood vessels (using OCT *en face* image), registering all time points to Day 8 OCT volume (see Supplementary Fig. S3). We then fine-tuned each registration using iterative closest point (ICP), computing the rigid-body transformation between CNS cell bodies’ centers based on our 3D CNS cell body mask (see Supplementary Fig. S3). To enable elastic registration, we applied a flexible ICP registration by dividing each volume into smaller sub-volumes of 50 μm^3^. Voxels containing > 10 cells were independently registered by ICP (see Supplementary Fig. S3). Fine registration was kept for voxels with less than 10 cells. Additionally, we applied quadratic smoothing between sub-volumes for a consistent smooth elastic transformation between volumes, to track temporal brain elasticity (Fig. 4a). Finally, we were able to graphically represent integrated temporal changes of the brain’s elasticity (Fig. 4a) attributed mainly to cell migration and negligible mechanical forces (at the few-micrometer scale), as previously described also in humans^44^. Our cortical elasticity map shows the delta of the rigid and elastic registration between Days 10 and 1, with arrows’ color signifies the directionality of each individual cell body, while its length represents the change in the cells’ central position (Fig. 4a). Color-coding of subtle spatial movement in 3D revealed that subregions of the cortical column move similarly, probably due to weak mechanical forces (Fig. 4a). To quantify brain tissue movement, we calculated the distances between the center position of two registered cell bodies when comparing rigid and elastic registration methods across X, Y, and Z axes between imaging Day 1 to Day 8, Day 1 to Day 10, and Day 8 to Day 10 (Fig. 4b–d). Moreover, we were able to quantify cell bodies’ volumes, density, and mean cell distance over time (see Supplementary Fig. S4).

## Discussion

Neuroimaging platforms are often costly, label-dependent, and limited in their penetrance depth, area size, and resolution. Improving temporal and spatial resolutions are among the greatest challenges of the field^45^. Here we demonstrate a novel OCT-based imaging technique coupled with a feature segmentation algorithm that has four major advantages: (1) Maintains high-resolution brain imaging using a commercial OCT device, while penetrating across cortical layers I-V down to 700 μm in depth of the mouse brain, (2) Enables high-resolution monitoring of spatial behavior of individual CNS cell bodies in a wide field of view, (3) Quantifies brain tissue movement by tracking individual CNS cell bodies location with time, and (4) Profiles complex morphology, such as CNS cell bodies’ density, volume, and average distance to the nearest cell throughout the cortical column in 3D.

Maintaining high-resolution brain imaging was achieved by developing a highly stable OCT setup. The resulting high-resolution images of the cortical layers were a pre-condition for developing our feature segmentation algorithm, acting as a filter. This filter was built with one layer of circular kernel convolution, as in convolutional neural network algorithm^39^. Then, a 3D CNS cell body mask was made by a series of 3D morphological thresholding. That 3D mask allowed us to quantify fine cellular and structural morphological features. Altogether, we achieved higher temporal resolution for longitudinal studies at micro-scale resolution. By imaging our 3D CNS cell bodies at the same ROI over time, we revealed the delicate temporal changes of the brain’s tissue (Fig. 4a). Although the presented OCT setup is applicable only to rodent models, it allows quantification of multiple morphological traits, such as CNS cell bodies’ density, tracing myelinated processes’ network and performing of longitudinal monitoring of subtle brain tissue movement at the same time. Hence, it may serve as a powerful approach for pre-clinical studies of brain injuries and neurodegeneration^34^, which involve temporal changes in brain morphology due to neuroinflammation and tissue atrophy.

Nevertheless, our study has several limitations. In spite of the supportive data acquired by others, showing that CNS cell bodies seem as circular dark foci in OCT images^27–29, 33–37, 46^, we are aware to the great importance of more comprehensive validation of these findings by histology. Therefore, we have dedicated a separate scientific effort to enable fine and accurate alignment between OCT to corresponding histology images. As this turns out to be a great technical challenge exceeding the scope of this study, we hope to soon officially introduce our OCT-to-Histology alignment tool^32^. Additionally, we observed intra- and inter-individual variabilities (Fig. 3b and Supplementary Fig. S4, respectively) in CNS cell bodies counts. These variabilities may stem from feature segmentation algorithm performance, or subtle changes in CNS morphology. Finally, we manually extracted CNS cell bodies average size in each cortical layer and used that parameter to set up our kernel size for the feature segmentation algorithm. Future studies could potentially automate this process to reduce cell body count variability.

Cutting edge neuroimaging techniques are starting to provide information on cell–cell interactions, while integrating spatial, temporal, and functional dimensions. Next generation OCTs may serve as compelling platforms pertinent to both basic biological questions and translational science. We show here that OCT can be used to provide high-resolution images of individual CNS cell bodies in vivo. Our modality and algorithm enable 3D imaging of complex tissue architecture and fine structures. Importantly, we were able to track the position of individual CNS cells over time, reflecting our system stability, and our ability to precisely align to the same volumes over the course of days. Finally, this platform can be highly transformative to quickly assess brain damage, including hemorrhages, axonal injuries, atrophy, and edema in rodent models. Given the implications of our results, our system can be of a major interest and importance to a wide audience across multiple scientific and medical disciplines.

## Materials and methods

### Animals

This study was conducted in accordance with National Institutes of Health (NIH) guidelines; protocols were approved by the Institutional Animal Care and Use Committee at Stanford University guidelines under protocols 33,146 and 27,499. C57/BL6 (8–12 weeks) male mice were housed for 2 weeks prior imaging in a 12-h light/12-h dark cycle with food and water available ad libitum. All methods were reported considering ARRIVE guidelines.

### Cranial window preparation

Cranial window installations were performed as previously described^31^. Briefly, mice were anesthetized with 5% isoflurane for induction and ~ 1–2% isoflurane during surgery. The skull was exposed, cleaned, and coated with a layer of Vetbond (3 M). A circular (1 cm diameter, 1 mm height) titanium implant with a counter bore (8 mm outer diameter and 6 mm through hole) was affixed to the skull with Metabond dental cement (Parkell) centered on − 2.75 mm (lateral) and − 2.25 mm (posterior) from bregma over the lateral portion of primary visual cortex of the left hemisphere. The mouse was transferred to a head clamp device designed to firmly hold the metal implant by an angled groove around its perimeter (this same head clamp device design was used to hold the animal under the OCT and thus had micron-level stability). A circular craniotomy was performed using a high-speed drill by slowly drilling away bone within the perimeter of the through hole of the implant. Once the bone was as thin as possible, but before drilling all the way through the bone, the remaining intact bone was pulled away with forceps to reveal the underlying cortex with the dura fully intact. Since these mice were initially intended for fluorescence imaging in a different project, not the subject of the present manuscript, injections of 0.5 µl of AAV8-CaMKIIα-GCaMP6m-p2A-ChRmine-Kv2.1 were performed with a 25 µm tip glass pipette (in vivo two photon imaging confirmed little to no fluorescence expression from the virus before transferring the mice to the OCT protocols). Afterward, a 4 mm glass coverslip affixed with UV-cured optical glue (Newport) to a titanium cannula of the same diameter (the cannula also had an ~ 8 mm flange at the top to register with the outer circular implant) was applied the surface of cortex and cemented in place with Metabond. For analgesia, buprenorphine sustained release (SR) was injected pre-operatively at 0.3–1.0 mg/kg subcutaneously, or buprenorphine (0.05–0.1 mg/kg) was injected by subcutaneous or intraperitoneal injections up to 72 h.

### Imaging system

All OCT images were acquired using a commercial spectral-domain OCT system (GAN220, Thorlabs, Newton, NJ), with a center wavelength of 900 nm and 200 nm bandwidth. The system was outfitted with a 20 × water immersed Olympus lens (UMPLFLN20XW), which provides a theoretical axial resolution of 2 μm (FWHM) in tissue, a theoretical lateral resolution of 0.76 μm (FWHM), an effective numerical aperture of 0.5, and ~ 11 μm depth of field. The OCT spectrometer acquires 2048 samples for each A-scan at a rate of 91 kHz. The OCT volumes were scanned in a field-of-view of 0.7 mm by 0.7 mm without significant signal loss at the edges of the scan.

### Imaging protocol

Mice were imaged approximately 9 months after the implantation of the cranial window. The mice were anesthetized with 2% isoflurane and placed in a custom head clamp device (under 2% isoflurane) that was originally designed for two-photon imaging with micron-level stability^31^, and was adapted to hold the animal under the OCT at the same conditions. The head-clamp was placed on an XYZ translation stage (Thorlabs MT3-Z8), moving in the z-direction only, for multiple volume imaging, with the interval between two consecutive focal plains set to 10 μm. The 10 μm step was optimized to minimize the scanning time while sampling tight enough (smaller than the effective Rayleigh length) in order to preserve high lateral resolution. As such, approximately 50 volumes were acquired in each imaging session, at 1 μm pixel resolution, using a refractive index of 1.33, and a field of view of 0.7 mm by 0.7 mm, resulting in a total of 1000 b-scans for each individual volume and a total imaging time of ~ 40 min for all volumes (~ 50,000 total b-scans). Three imaging sessions were completed at three different days: Day 1, Day 8, and Day 10.

### Feature segmentation algorithm

All brain volume processing and further analysis were performed with Matlab (MathWorks, Natick, MA). Visualization of brain volume and individual slices was done using ImageJ^47^. The feature segmentation algorithm is based on one-layer convolutional neural network principles as follows: (1) individual *en face* slices were convolved with a circular kernel (2) a 99.5% value threshold was applied to each individual *en face* from step 1. (3) 3D morphological thresholding was applied to the entire volume to reconstruct a 3D CNS cell body mask, under three conditions: (a) A cell body must appear in at least three consecutive axial slices, meaning having a minimum depth of 4 µm, and a maximum depth equal to its diameter. (b) Cell body’s volume should not exceed the volume of the same radius sphere, and (c) its axial spread should not exceed twice its diameter. Our feature segmentation algorithm, which acts as a classifier, identifies a CNS cell body with 81% accuracy (or PPV, see Supplementary Fig. S5), meaning a low false discovery rate. However, our classifier only captures 54% of the cells between two or more time points (low sensitivity). Source code openly available at: https://github.com/lautman/OCT2BrainSegmentation.

### Iterative closest point registration algorithm

To register the three time points together and track the same CNS cell bodies, we applied a rigid-body registration, followed by an elastic one. The first step was applying a course registration scheme, using Matlab (MathWorks, Natick, MA) by matching blood vessels (using OCT *en face* image). All three time points were registered to the Day 8 OCT volume (Day 1 to Day 8, and Day 10 to Day 8, see Supplementary Fig. S3). The second step was to fine-tune each registration using Iterative Closest Point (ICP) method, computing the rigid-body transformation between CNS cell bodies’ centers (based on our 3D CNS cell body mask, see Supplementary Fig. S3), assuming that registered cells are less than 10 µm apart. The final step was about enabling elastic registration, in the form of a flexible ICP registration. We divided each volume into smaller sub-volumes of 50 μm^3^ and registered independently each one, using ICP, only if a voxel was containing > 10 cells (see Supplementary Fig. S3), else we kept the fine registration as is. Registered cells were less than 10 µm apart. Additionally, we applied quadratic smoothing between sub-volumes for a consistent smooth elastic transformation between volumes, to track temporal brain’s elasticity (Fig. 4a). An example result of the elastic registration can be seen in Supplementary Fig. S5, where a Venn diagram represents the CNS cell bodies segmented in each day and the resulting registration with the other days.

## Supplementary Information

Supplementary Information.

## Data Availability

The datasets used and analyzed during the current study available from the corresponding author on reasonable request. Source code openly available at: https://github.com/lautman/OCT2BrainSegmentation.
