# Supplementary material for: Intravital 3D visualization and segmentation of murine neural networks at micron resolution
Source: Sci Rep. 2022 Jul 30;12:13130. doi: 10.1038/s41598-022-14450-0 (PMC9338956; doi:10.1038/s41598-022-14450-0)
Supplement: Supplementary file 1 — Supplementary Information. [file 41598_2022_14450_MOESM1_ESM.pdf]

Intravital 3D visualization and segmentation of murine neural networks at micron resolution

Ziv Lautman[1,2,3*,] Yonatan Winetraub[1,3,4,5*], Eran Blacher[6,7*], Caroline Yu[1,3], Itamar Terem[1,3,8], Adelaida Chibukhchyan[2], James H. Marshel[9], Adam de la Zerda[1,3,4,5,8,10 †]

[1]Department of Structural Biology, Stanford University School of Medicine, Stanford, CA, 94305, USA.

[2]Department of Bioengineering, Stanford University, Stanford, CA, 94305, USA.

[3]Molecular Imaging Program at Stanford, Stanford, CA, 94305, USA.

[4]Biophysics Program at Stanford, Stanford, CA, 94305, USA.

[5]The Bio-X Program, Stanford, CA, 94305, USA.

[6]Department of Neurology & Neurological Sciences, Stanford School of Medicine, Stanford, CA, 94305, USA.

[7]Department of Biological Chemistry, The Alexander Silberman Institute of Life Sciences, The Hebrew University of Jerusalem, Edmond J. Safra Campus Givat-Ram, Jerusalem 9190401, Israel.

[8]Department of Electrical Engineering, Stanford University, Stanford, CA 94305, USA

[9]CNC Department, Stanford University, Stanford, CA 94305, USA.

[10]The Chan Zuckerberg Biohub, San Francisco, CA, 94158, USA.

*These authors contributed equally: Ziv Lautman, Yonatan Winetraub, and Eran Blacher

†Corresponding author: Adam de la Zerda
Email: adlz@stanford.edu

Supplementary Information

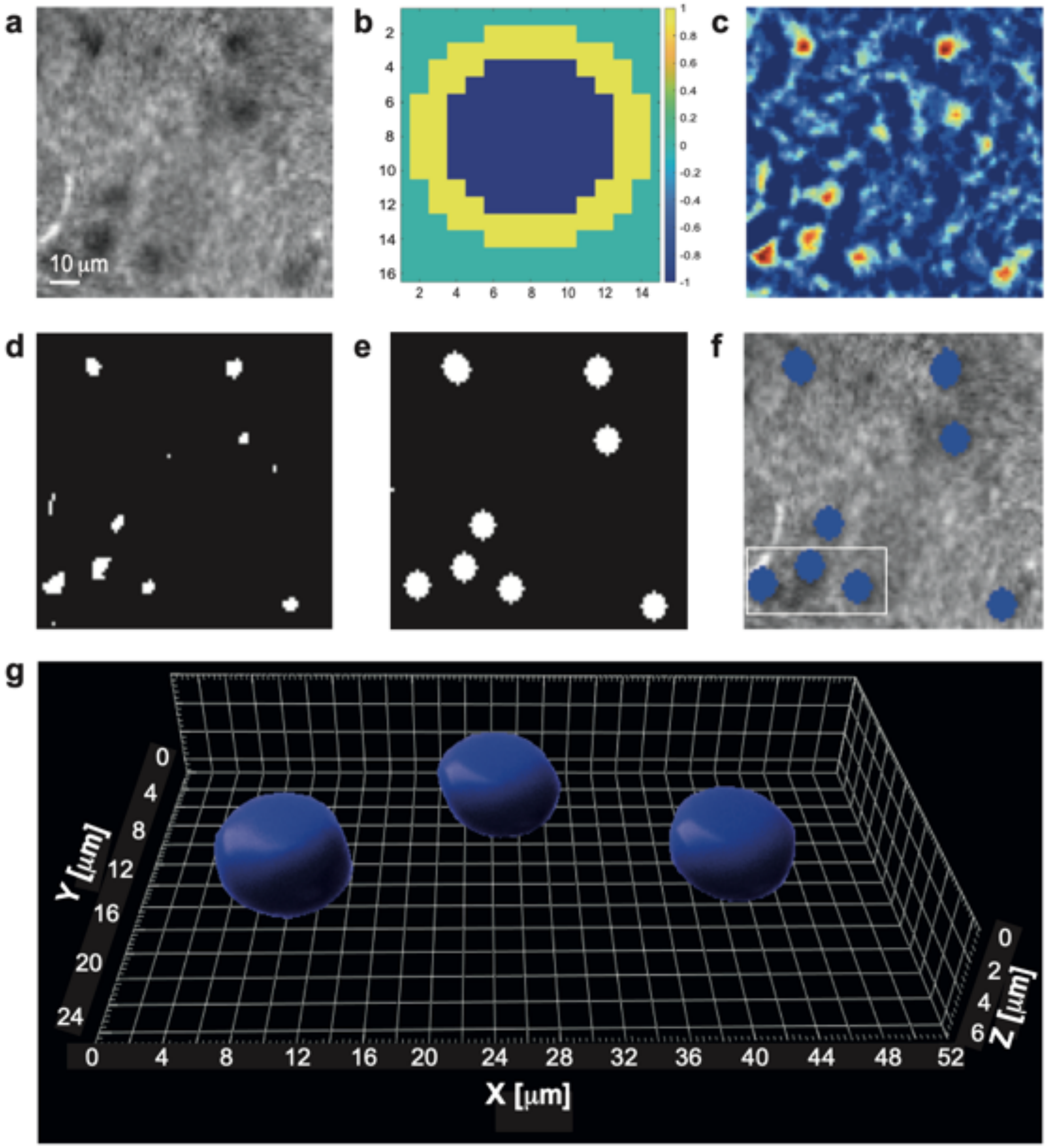

Supplementary Figure S1. Feature segmentation algorithm. (**a**) an example of *en face* OCT image representing 8 cell bodies that appear as circular dark foci. (**b**) The circular 8 µm kernel used by the algorithm. (**c**) A heat map of convolution of (a) with (b). "Hot spots" represent CNS cell bodies. (**d**) Applying 99.5% value threshold to (c). (**e**) 3D morphological thresholding, applied to the entire imaging volume after each individual *en face* processed as in (a-d). (**f**) Final image of the feature segmentation algorithm overlaid

on top of (a). (**g**) 3D visualization of the CNS cell bodies highlighted in the white frame in (f)

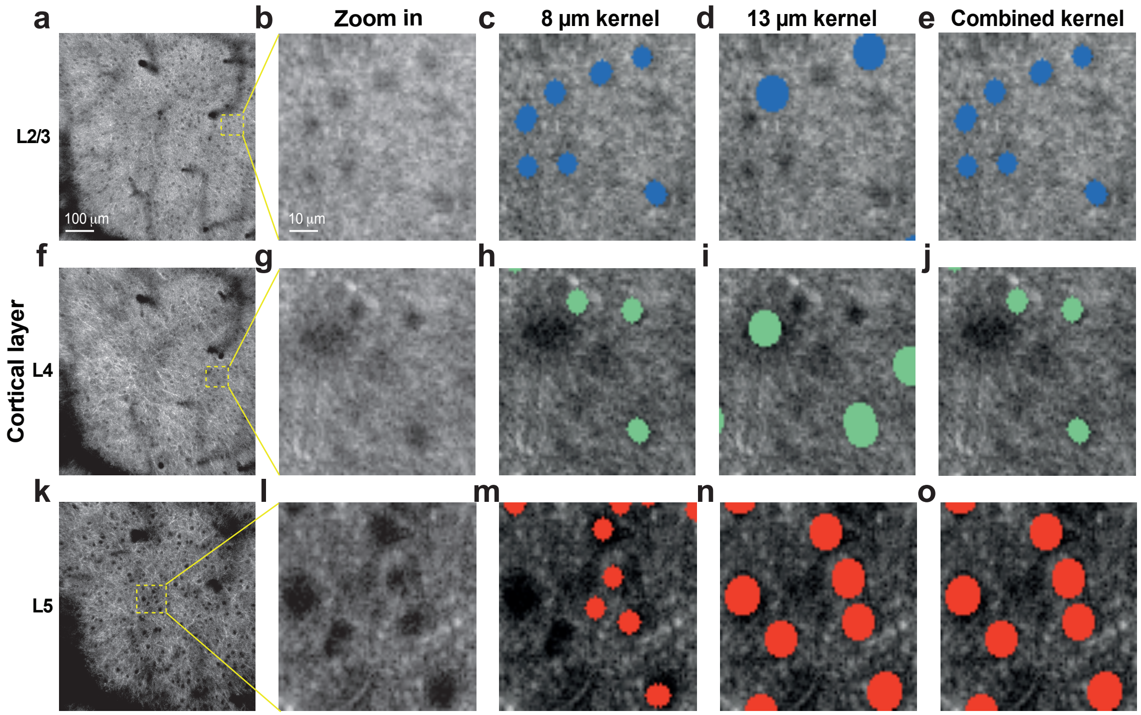

Supplementary Figure S2. Fine tuning the kernel for the feature segmentation algorithm. (**a-e**) Cortical layer 2/3 segmentation indicates that an 8 µm kernel is optimal. (**f-j**) cortical layer 4 segmentation indicates that an 8 µm kernel is optimal. (**k-o**) Cortical layer 5 segmentation indicates that a 13 µm kernel is optimal. The combined kernel consists of an 8 µm kernel for layers 2/3/4 and a 13 µm for layer 5.

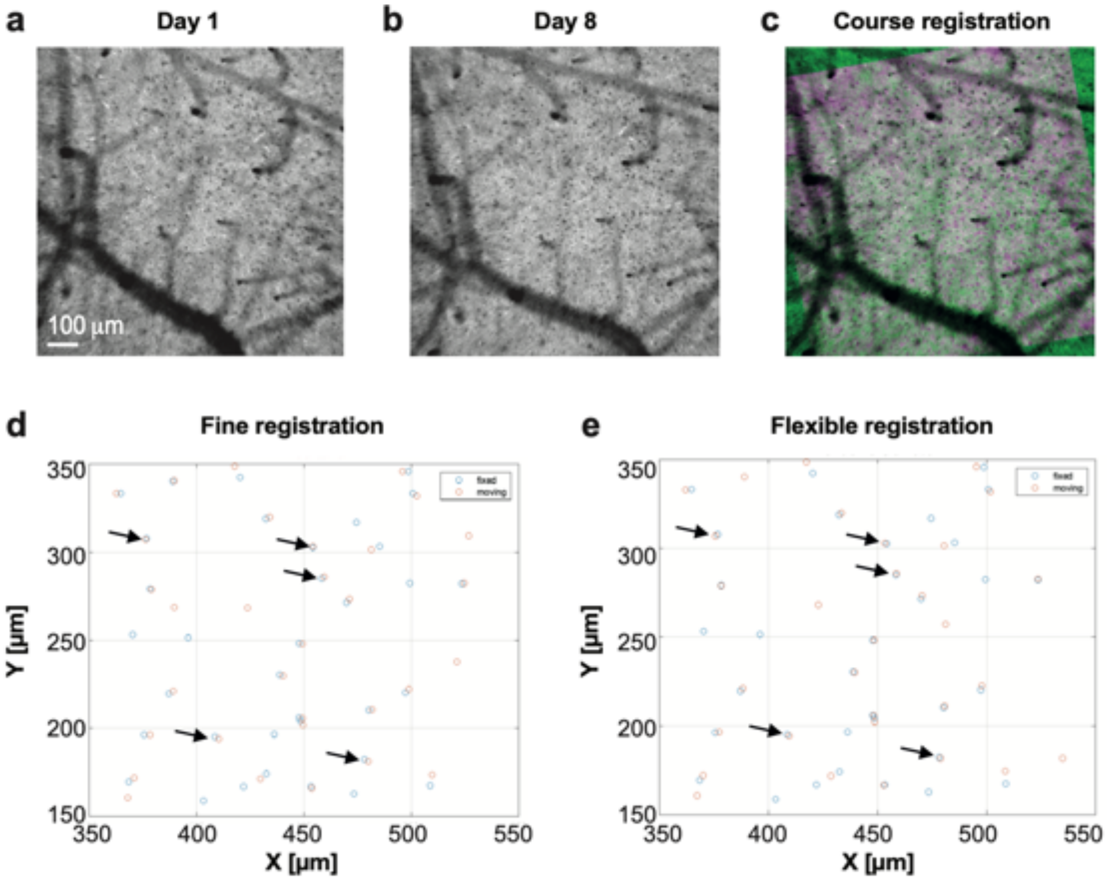

Supplementary Figure S3. Registration of the same region of interest (ROI) over time. (**a-c**) Course registration of Day 1 OCT volume to Day 8 OCT volume, by matching blood vessels of a sampled *en face* at approximately the same depth. (**d**) Magnification of a sampled CNS cell body mask of a single *en face*, after fine registration using Iterative Closest Point (ICP). Fixed (blue) cells represent Day 8 CNS cell bodies, while moving (red) cells represent Day 1 CNS cell bodies. (**e**) Same Magnification of a sampled CNS cell body mask of a single *en face* as panel e, after a flexible (elastic) registration. Arrows (d-e) indicate the difference in cells' registration between the two methods.

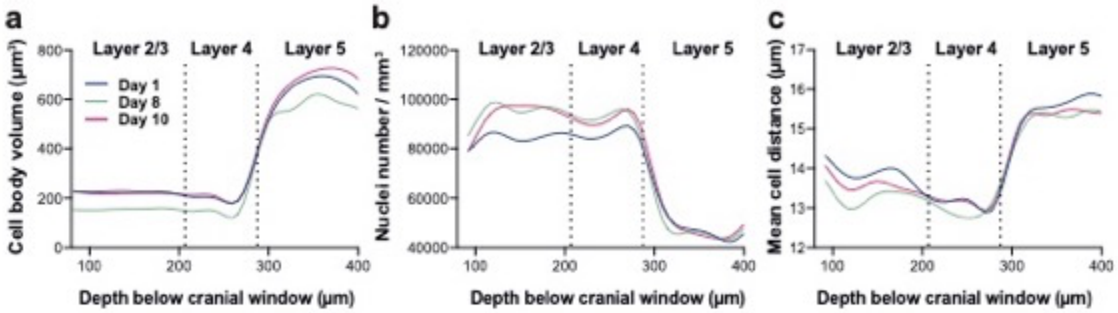

Supplementary Figure S4. Quantifying cellular morphological features across the cortical column of the same mouse over time. The same mouse was longitudinally imaged by our OCT neuroimaging system and morphological traits were assessed and quantified: (**a**) average CNS cell body volume (**b**) average CNS cell body density (**c**) average distance to the nearest neighboring CNS cell body.

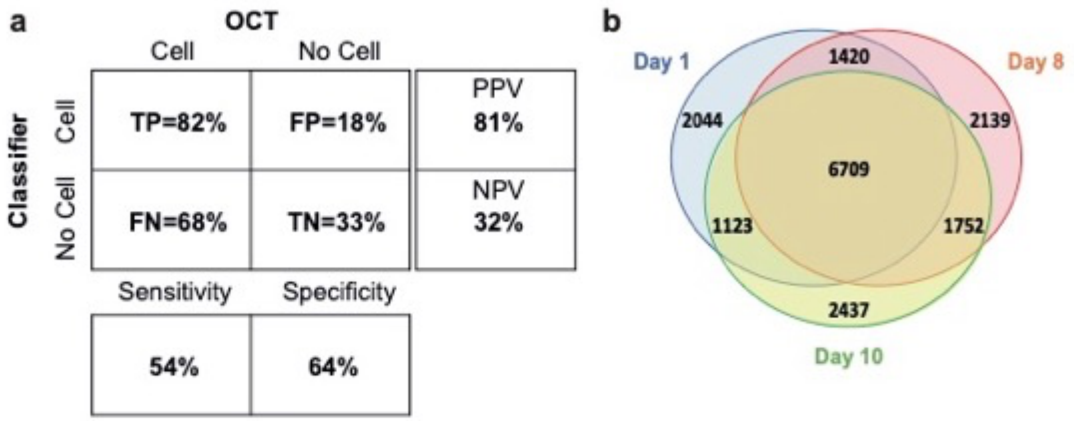

Supplementary Figure S5. Feature segmentation algorithm's performance. (**a**) The confusion matrix of the feature segmentation algorithm. (**b**) A Venn diagram representing the CNS cell bodies segmented in each imaging session (OCT volume) and the resulting registration with the other sessions (Days 1, 8, and 10).

Supplementary media

Supplementary Media 1: https://youtu.be/0BdD2_PkWks

Deep OCT volumetric imaging of murine visual cortex at a cell-scale resolution, up to 700 μm deep and across a large field of view of up to 0.7 x 0.7 mm. CNS cell bodies appeared as circular dark foci, while myelinated axons processes manifest as white ramifications protruding across the cortical layers

Supplementary Media 2: https://youtu.be/DS5gO4rd2h0

3D segmentation of CNS cell bodies and myelinated processes from deep OCT volumetric imaging of murine visual cortex at a cell-scale resolution.
